# High prevalence of muscle stability deficits in semi‐professional football players—Adaptive Force assessment reveals selective impairment of holding capacity with preserved maximal strength: A cross‐sectional study

**DOI:** 10.1002/ksa.70525

**Published:** 2026-07-07

**Authors:** Laura V. Schaefer, Frank N. Bittmann, Jaali Ulrich, Robert Prill, Roland Becker

**Affiliations:** ^1^ Centre of Orthopaedics, Traumatology and Plastic Surgery University Hospital Brandenburg, Brandenburg Medical School Theodor Fontane Brandenburg an der Havel Germany; ^2^ Faculty of Health Sciences Joint Faculty of University of Potsdam, Brandenburg Medical School Theodor Fontane and Brandenburg Technical University Cottbus‐Senftenberg Brandenburg Germany; ^3^ Regulative Physiology and Prevention, Department of Sports and Health Sciences University of Potsdam Potsdam Germany

**Keywords:** Adaptive force, Football injuries, muscle function assessment, muscle stability, neuromuscular control

## Abstract

**Purpose:**

Despite considerable research, strength screening has shown limited value for injury prediction. The concept of muscle stability assessed by Adaptive Force (AF) offers a distinct perspective—addressing neuromuscular holding capacity closer to injury‐prone motions rather than pushing strength. This study aimed to assess the prevalence and distribution of muscle stability deficits in football players and their discriminative capacity relative to conventional strength parameters.

**Methods:**

AF and maximal voluntary isometric contraction (MVIC) were measured in 23 male semi‐professional football players. Five muscle groups were tested bilaterally (knee extensors/flexors; hip flexors/adductors/abductors). AF was assessed by objectified manual muscle tests with a handheld device recording force and angular velocity. AF parameters, MVIC and the conventional hamstrings‐to‐quadriceps (H:Q) ratio were compared between stability categories.

**Results:**

Stability deficits were highly prevalent: 83% of players had at least one deficit; 31% of all tested muscle groups were unstable, highest in hip abductors (52%) followed by hamstrings (46%). The maximal holding capacity was selectively impaired in unstable muscles (on average 61% lower than the maximal force output), while MVIC and maximal AF did not differ between stability categories. The ratio of holding capacity to maximal AF (AF‐Ratio) was the strongest discriminator (stable vs. unstable: *d* = 6.62, *ω* ^2 ^= 0.88), largely consistent across muscle groups and uncorrelated with the H:Q ratio. The H:Q ratio failed to discriminate between stable and unstable hamstrings (*d* = −0.04), whereas the AF‐Ratio discriminated strongly (*d* = 6.43).

**Conclusion:**

Muscle instability represents a selective impairment of holding capacity with preserved maximal strength, not detected by MVIC testing. By capturing a distinct neuromuscular function, AF offers a novel mechanistic framework beyond pushing strength. Further studies in larger samples and prospective designs are required to assess the potential utility of stability assessment in clinical practice.

**Level of Evidence:**

Level III, diagnostic studies.

**Abbreviations**
ABD: hip abductors
ACL: anterior cruciate ligament
ADD: hip adductors
AF: Adaptive Force
AFiso_max_
: maximal isometric Adaptive Force (maximum holding capacity)
AF_max_
: maximal Adaptive Force
AF‐Ratio: ratio of AFiso_max_ to AF_max_ (stability quotient)
ANOVA: analysis of variance
HAM: hamstrings (knee flexors)
HFL: hip flexors
HIMA: holding isometric muscle action
H:Q ratio: hamstrings‐to‐quadriceps ratio
MMT: manual muscle test
MQF: quadriceps femoris muscle (knee extensors)
MVIC: maximal voluntary isometric contraction
PIMA: pushing isometric muscle action
RFD: rate of force development

## INTRODUCTION

Injuries constitute a substantial burden in football (soccer), with the lower extremity predominantly affected; muscles are the most frequently injured tissue, while ligament‐joint injuries account for the highest burden [23, 26]. The majority of injuries occur without direct contact [14, 15, 17, 23]. Despite considerable research efforts and preventive interventions, the incidence of hamstring (HAM) strain injuries doubled from 2001 to 2022 in elite professional football [16] and rates of non‐contact knee ligament injuries remain high [2, 23]. Reviews have found that strength measures, including strength ratios, offer limited predictive validity for HAM or anterior cruciate ligament (ACL) injuries [18, 19, 21, 39], with none of 13 strength‐related variables reaching significance in a meta‐analysis of 78 prospective studies [19] and insufficient evidence to recommend strength screening in professional football [3, 4, 18, 23, 40, 41]. This raises the question whether conventional strength assessment captures the neuromuscular qualities relevant to injury risk.

Conventional strength assessments measure the capacity to exert force against an external resistance, whether by maximal voluntary isometric contraction (MVIC)—recently termed pushing isometric muscle action (PIMA) [25, 28]—or isokinetic testing. However, non‐contact injuries occur precisely in moments when active muscles must adapt to external loads—during landing, turning, cutting and similar movements [14, 23, 27]—motor tasks that include active deceleration (eccentric muscle action) and stabilization [16, 27]. The latter includes holding isometric muscle action (HIMA), recently suggested to represent a more complex neuromuscular control strategy than PIMA [25, 28, 29].

The Adaptive Force (AF) concept provides a framework for assessing the neuromuscular capacity to adapt to varying external loads during HIMA, representing muscle stability [7, 8, 13, 30, 31, 33, 34, 35, 36, 37]. The maximal holding capacity represents the highest force level at which an individual can maintain static position during an increasing external load; if this is exceeded, the muscle yields into eccentric muscle action. Previous research has shown that the holding capacity is particularly sensitive to proprioceptive irritation [7, 8], negative emotions [34, 35, 37] and post‐infectious states [31, 32] by showing instant reductions of 39%–53% relative to peak force, suggesting the involvement of central regulatory mechanisms. These findings indicate that the holding capacity captures aspects of neuromuscular function not assessed by traditional strength testing.

Despite this rationale, no study has systematically investigated lower extremity muscle stability assessed by AF in a football population. Given the high prevalence of injuries and the gaps in current evidence of strength‐based approaches, such investigation appears warranted.

The aims of this exploratory cross‐sectional study were (1) to assess the prevalence and distribution of muscle stability deficits in the lower extremities of competitive semi‐professional football players, (2) to compare force parameters between stability categories and (3) to evaluate the holding capacity as a muscle‐independent stability parameter. Based on previous findings and the proposed neurophysiological distinction between HIMA and PIMA, it was anticipated that stability deficits would manifest selectively in the holding capacity while leaving conventional strength parameters unaffected.

## MATERIALS AND METHODS

This cross‐sectional study was conducted during the preseason preparation period in July/August 2025 of a semi‐professional football team, which plays in the sixth tier of the German football league system. The testing session (~75 min) was performed at the team's training facility prior to training.

### Participants

Twenty‐three male semi‐professional football players volunteered to participate (age: 23.7 ± 5.7 years [range: 18–39], height: 184.4 ± 5.6 cm [174–195], mass: 79.3 ± 6.9 kg [66–94], training: 6.3 ± 1.0 h/week [4–9] [excluding matches; 11 players reported additional fitness training: 1–6 times/week]; leg preference: 5 left, 15 right, 3 bilateral). Inclusion criterion was active participation in regular football training in the first team (≥3 sessions per week). Exclusion criteria were acute injury precluding participation in testing, and neurological conditions affecting motor control. Based on previous AF studies reporting very large effect sizes (*d* = 2.72) [30], an a priori power analysis indicated that the available team size of 23 players was adequate.

### Technical equipment for AF and MVIC assessment

**Figure 1:**
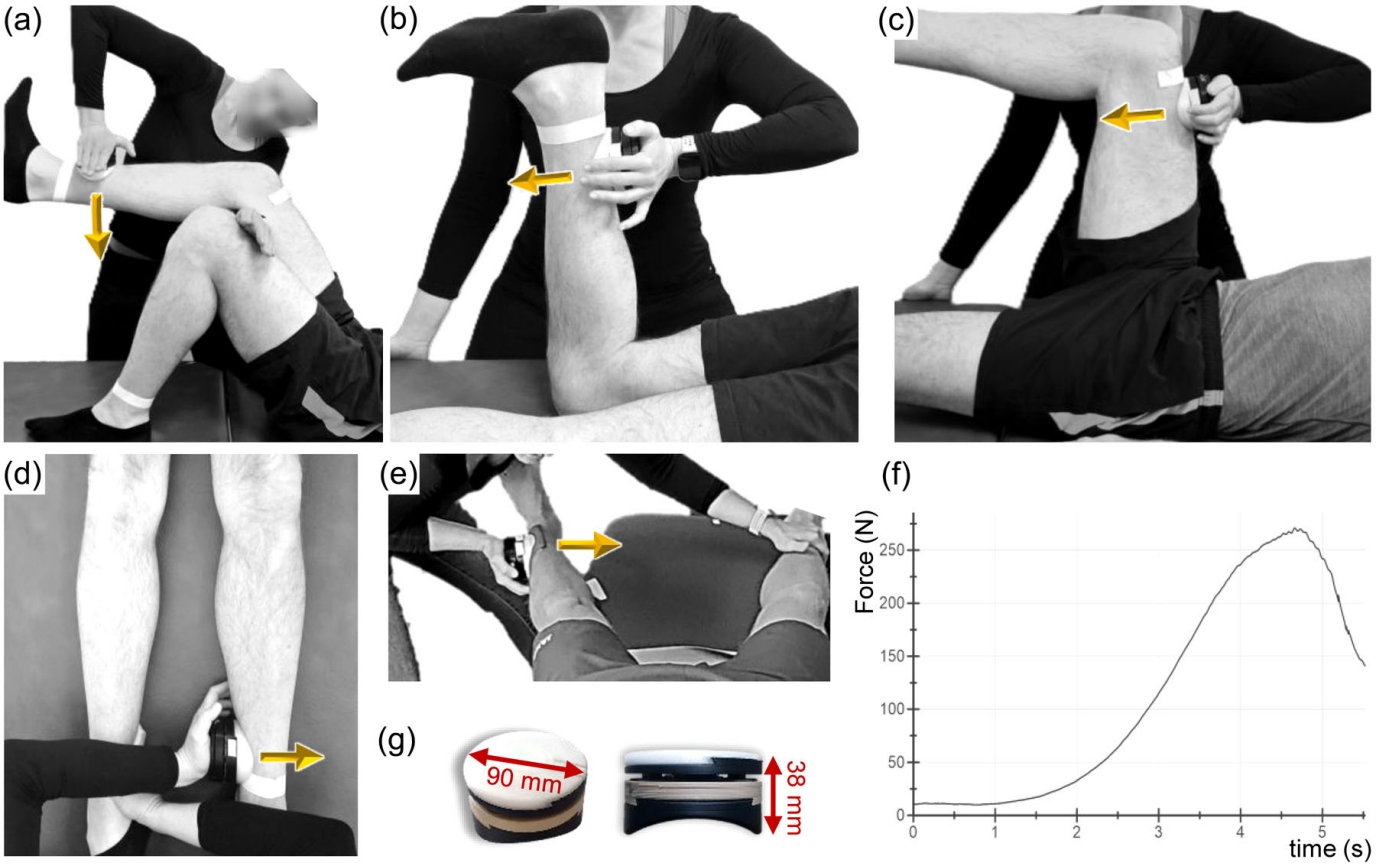
Starting positions of tested muscles and schematic force profile of AF trials. The starting position and force vector applied by the examiner correspond to the plane of the main function of the muscle group being tested (a) MQF–knee extension, (b) HAM–knee flexion, (c) HFL–hip flexion, (d) ADD–hip adduction and (e) ABD–hip abduction. For AF measurements, the examiner applied increasing force in the direction of muscle lengthening (arrow direction), following a standardized force profile (f), with the maximum force depending on test muscle and examiner–participant constellation. The handheld device (g) was placed between the examiner's palm and the participant's limb and measured force and position during the test. The same starting positions were used for MVIC tests, but the participant pushed against the fixed resistance provided by the examiner (isometric activation in direction of muscle shortening; arrow direction not shown). ABD, hip abductors; ADD, hip adductors; AF, Adaptive Force; HAM, hamstrings; HFL, hip flexors, MQF, quadriceps femoris muscle; MVIC, maximal voluntary isometric contraction.

AF was assessed using an objectified manual muscle test (MMT) with a dedicated handheld device (Figure 1g) incorporating strain gauges (precision: 1.0 ± 0.1%) and kinematic sensors (Bosch BNO055, nine‐axis absolute orientation sensor) for simultaneous recording of force and change of position (sampling rate: 180 Hz). Data were transmitted via Bluetooth to a tablet. MVIC was also recorded by the handheld devices, except for quadriceps femoris muscle (MQF) due to high forces involved, where MicroFET devices were used (Hoggan, MicroFET2, MicroFET3), which can only measure peak force.

### Setting and testing procedure

The measurements were executed by two experienced examiners (male and female; MMT experience: 30 and 14 years, respectively). Participants were assigned to examiners based on recruitment order. Five muscle groups were measured bilaterally (10 per participant): MQF (knee extension), HAM (knee flexion), hip flexors (HFL, primarily targeting rectus femoris muscle due to test positioning), hip adductors (ADD) and hip abductors (ABD). To minimize the influence of the side measured first, the starting side was randomized (left: 10 participants, right: 13 participants). For all tests, participants lay supine (MQF, HFL, ADD, ABD) or prone (HAM) on an examination table (Figure 1a–e).

After the introduction, 12 MMTs without handheld device were performed to familiarize the players with the test execution and to obtain an overview of the holding ability in general (bilaterally tested muscle groups: HFL, ADD, ABD, HAM, pectoralis major and deltoideus muscles).

The MVIC tests followed (2× per side/muscle; resting period: 60 s). The participant had the task to push with maximum force against the device placed in the palm of the examiner, who provided a fixed resistance (smooth force increase (2–3 s), maintain maximum for ~1 s). For the MQF test, a fixed loop was tied around the examination table with the device (MicroFET) installed since the strength was assumed to exceed the examiner's resistance. Standardized starting positions were set (Figure 1); marked positions and lever length assured the reproducibility for the subsequent AF tests.

The AF assessment (3× per side/muscle; resting period: 30 s) used objectified MMTs with handheld device in identical starting positions as in MVIC tests; however, the procedure differed significantly. The participant's task was to maintain the starting position (isometric hold) while adapting to the progressively increasing force applied by the examiner on the participant's limb. The force application followed a standardized profile (Figure 1f), previously described in detail [6, 34]. If the participant was able to maintain position throughout the force increase, the test was rated as ‘stable’ by the examiner. If the limb yielded during force application (involuntary transition from isometric to eccentric muscle action), the test was rated as ‘unstable’. Muscles that could not be definitively categorized as stable or unstable were classified as ‘borderline’. Examiners were not blinded to previous trials, as tactile perception of muscle behaviour during force application is inseparable from test execution. Reproducibility of the force profile application of both examiners has been demonstrated previously, with high agreement of the standardized force profile within and between examiners (intraclass correlation, ICC(3,1) = 0.989) [6].

### Data processing and statistical analysis

#### Data processing and parameter extraction

**Figure 2:**
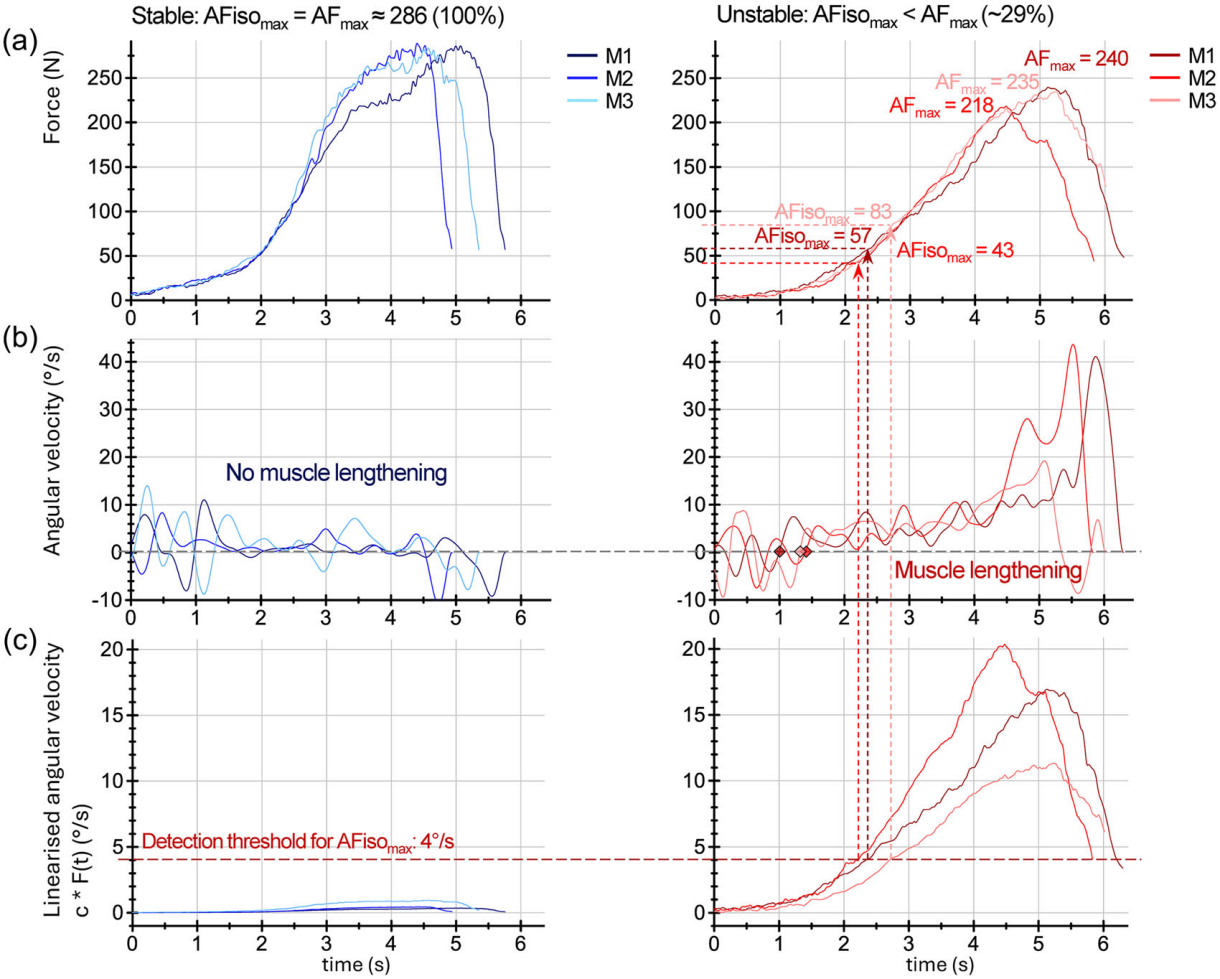
Exemplary curves of force (N, a), angular velocity (°/s, b) and linearized angular velocity (°/s, c) for three consecutive AF trials (M1–M3) of knee flexors (HAM) of two different players. Left panels: Stable muscles—angular velocity oscillates around zero throughout (b), and linearized angular velocity (c) remains below the detection threshold for AFiso_max_ (horizontal dashed line), indicating that the starting position was maintained quasi‐stable until peak force; AFiso_max_ = AF_max_ (100%). (Player A, right HAM, MVIC: 297 N; male examiner). Right panels: unstable muscles—diamond markers in (b) indicate zero‐crossings representing the onset of muscle lengthening. The linearized angular velocity (c) exceeds the detection threshold during force increase, marking AFiso_max_ (vertical dashed lines); AFiso_max_ is substantially lower than AF_max_ (~29%). (Player B, left HAM, MVIC: 220 N; female examiner). (b) Angular velocity: gyroscope signal (gX axis), low‐pass filtered (Butterworth, 2 Hz, fourth order); dashed horizontal line indicates zero. (c) Linearized angular velocity: c · F(t), where c is the slope of the least‐squares fit (see Methods). In the absence of muscle lengthening, c approximates zero and the linearized angular velocity remains near zero despite increasing force; since c is a single scalar, c · F(t) necessarily follows the shape of the force curve. The similar force increases reflect reproducible force application of both examiners. AF, Adaptive Force; AFiso_max_, maximal isometric AF; AF_max_, maximal AF; HAM, hamstrings; MVIC, maximal voluntary isometric contraction.

Data were processed using NI DIAdem 2019 (National Instruments) and Python 3.12 (Python Software Foundation). Raw signals were interpolated (linear spline) to obtain equidistant time channels (1000 Hz) and filtered (Butterworth low‐pass, cut‐off frequency 20 Hz, filter degree 5). Torque was calculated as force (N) × lever length (m) to enable standardized comparisons across limbs and participants. The following parameters were extracted:

(1) MVIC (Nm): Peak value of MVIC trials (maximum of two trials was used for evaluation).
(2) AF_max_ (Nm): Peak value of AF trials; may occur during isometric or eccentric muscle action.
(3) AFiso_max_ (Nm): Highest force value maintained in static starting position of AF trials (maximum holding capacity). The position of the limb must necessarily be considered. If it remains quasi‐stable until the peak force, AFiso_max _= AF_max_. If the limb begins to yield during the force increase, the corresponding force value represents AFiso_max_. The gyroscope signal (gX) was used to assess the change of limb position throughout the trial. A least‐squares linear fit without intercept (gX = c · F) was applied to gX (°/s) and force signal F (N) over the range from force onset to end of measurement (defined as 20% AF_max_ in descending phase; where a substantial force drop with subsequent slight recovery occurred, the endpoint was set at the corresponding force minimum). The slope coefficient c [(°/s)/N] represents the rate of sensor rotation per unit of applied force. AFiso_max_ was determined as the force at which c · F(t) first exceeded a defined muscle‐specific threshold: HFL, MQF, HAM: 4 °/s; ADD, ABD: 2 °/s (Figure 2). The thresholds were chosen within the quasi‐isometric range, accounting for the smaller test range of motion in ADD/ABD. The resulting ratio of AFiso_max_ to AF_max_ reflects the expected pattern from prior research [30]: ~100% in clinically stable trials and substantially reduced in clinically unstable trials. Robustness against alternative threshold definitions was confirmed by sensitivity analyses (see Supporting Information S1: Section 2). The algorithmically determined AFiso_max_ approximates the true force value at the start of muscle lengthening (break point) and is used as the operational definition of AFiso_max_ in all subsequent analyses.
(4) AF‐Ratio (%): AFiso_max_ divided by AF_max _× 100 (stability quotient).
(5) H:Q ratio (%): MVIC of HAM divided by MVIC of MQF × 100 (conventional hamstring‐to‐quadriceps strength ratio [5, 18]).

For statistical comparisons, AF parameters were averaged across available trials per muscle group and side, yielding one value per parameter at muscle group level (*n* = 230; 5 muscle groups × 2 sides × 23 players).

#### Prerequisites for statistical analysis

##### Stability classification

Stability was classified automatically using a gyroscope‐based algorithm [see Supporting Information S1: Section 1 for classification rules, muscle‐specific boundaries (Supporting Information S1: Table 1), validation and sensitivity analysis across different boundaries (Supporting Information S1: Table 3)]. Agreement between the algorithm and the examiners' ratings for individual trials (*n* = 685) and aggregated at muscle group level (*n* = 230) was almost perfect (individual: binary stable/unstable accuracy: 98.7%, weighted *κ* = 0.97; aggregated: 98.5%, weighted *κ* = 0.96; with no misclassifications between stable and unstable; Supporting Information S1: Table 2). A sensitivity analysis across alternative classification boundaries confirmed the robustness of the AF‐Ratio group differences (Supporting Information S1: Section 1.2). The algorithm classification was used for the subsequent analyses.

##### Rate of force development (RFD)

To verify comparable force application across groups, the RFD (linear regression on force signals from onset to 50% AF_max_; representing the phase of active force build‐up before considerable yielding effects) was compared between stability categories. No significant difference was found (*z*‐standardized, *p* = 0.514), confirming that observed differences in stability parameters are not attributable to differences in force application rate.

#### Statistical analysis

Descriptive statistics are presented as arithmetic mean, standard deviation (*M* ± SD) and 95% confidence intervals (CIs; in square brackets). Where continuous variables were compared across stability categories by pooling all muscle groups, values of absolute force parameters were *z*‐standardized within each muscle group to account for differences between muscles.

##### Stability classification and prevalence

Stability classifications were aggregated at muscle group level (*n* = 230; see Supporting Information S1: Section 1). Differences in instability rates between muscle groups were tested using Cochran's *Q* test, with post hoc McNemar tests (Bonferroni‐corrected for 10 comparisons); side differences were assessed using McNemar tests.

##### Force parameter comparisons

AFiso_max_, AF_max_, AF‐Ratio and MVIC were compared between stability categories (stable, borderline, unstable) at muscle group level (*n* = 230) using Welch's analysis of variance (ANOVA), which does not assume equal variances and is robust against moderate deviations from normality; effect size omega squared (*ω* ^2^) was calculated. Post hoc pairwise comparisons used Games‐Howell tests. For individual muscle groups, planned comparisons of stable versus unstable were performed using Welch's *t* tests, Bonferroni‐adjusted for five muscle groups. Additionally, muscle‐specific differences within each stability category were evaluated by Welch's ANOVA. To compare force parameters within the same muscle group and each stability category, repeated measures ANOVA was used to compare MVIC (PIMA), AFiso_max_ (HIMA) and AF_max_. Greenhouse‐Geisser corrected degrees of freedom are reported where the assumption of sphericity was violated, with partial eta squared (*η_p_* ^2^) as effect size. Post hoc pairwise comparisons used paired *t* tests, Bonferroni‐adjusted for three comparisons. Correlations between AFiso_max_, AF_max_ and MVIC were calculated separately within stable and unstable muscle groups using Pearson's correlation coefficients; differences between correlations were tested using Fisher's *z*‐transformation.

The conventional H:Q ratio was analysed using Welch's *t* tests between limbs classified as stable versus unstable by the examiners' clinical rating of HAM (limb level, *n* = 46). The same comparison was performed for the AF‐Ratio, both averaged across HAM and MQF and for HAM alone, to allow direct comparison of discriminative capacity. The examiners' rating served as an independent clinical criterion, as it was based on observation during testing and not derived from the recorded data. Correlation of H:Q ratio and AF‐Ratio was assessed using Spearman's rank correlation.

Cohen's *d* was used as effect size for all pairwise comparisons (*d* = 0.2, 0.5 and 0.8 interpreted as small, medium and large effects, respectively) [10]. Corresponding 95% CIs are given in square brackets. Significance was set at *p* < 0.05. Analyses were performed using Python 3.12 and jamovi 2.7.18. The large language model (Claude, Anthropic) was used to assist with data processing scripts, manuscript preparation and language refinement. All scientific content and interpretation remain the responsibility of the authors.

## RESULTS

Of 690 planned individual measurements (3 trials × 230 muscle groups), 685 were recorded; five were not assessed (four due to cramping, one due to a recording error).

### Distribution of muscle stability

**Figure 3:**
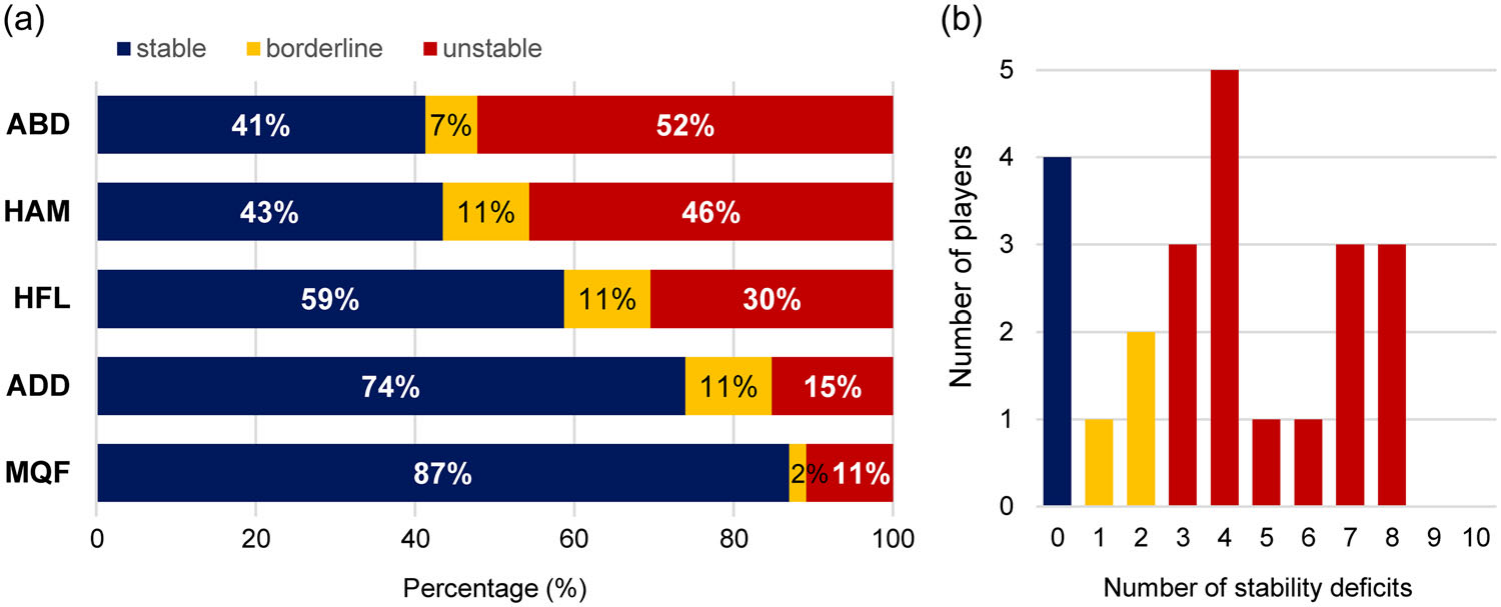
Distribution of muscle stability. (a) Stability classification per muscle group (*n* = 46: 23 players × 2 sides, aggregated across trials). (b) Number of stability deficits at muscle group level per player (aggregated; unstable or borderline; *n* = 23; maximum possible: 10 = 5 muscle groups × 2 sides). Dark blue: no deficits (completely stable); gold: 1–2 deficits; red: ≥ 3 deficits. *Note*: Seven muscle groups of six players classified as stable at the aggregated level showed isolated non‐stable trials (all single borderline classifications). ABD, hip abductors; ADD, hip adductors; HAM, hamstrings; HFL, hip flexors; MQF, quadriceps femoris muscle.

Of 230 muscle groups, 140 (61%) were classified as stable, 71 (31%) as unstable and 19 (8%) as borderline (Figure 3a). Instability rates differed significantly between muscle groups (*Q* = 20.7, *p* < 0.001); ABD showed the highest instability rate (52%), while MQF showed the lowest (11%). Bonferroni‐corrected post‐hoc comparisons confirmed significant differences between MQF and ABD (*p*_adj_ = 0.010) and between ADD and ABD (*p*_adj_ = 0.039). All other pairwise comparisons were not significant. No significant side differences were observed for any muscle group (all *p *> 0.37). At least one stability deficit was present in 34 of 46 limbs (74%) and 19 of 23 players (83%; Figure 3b). Four players (17%) were completely stable across the five bilaterally tested muscle groups (aggregated level).

### Force parameters by stability categories

**Figure 4:**
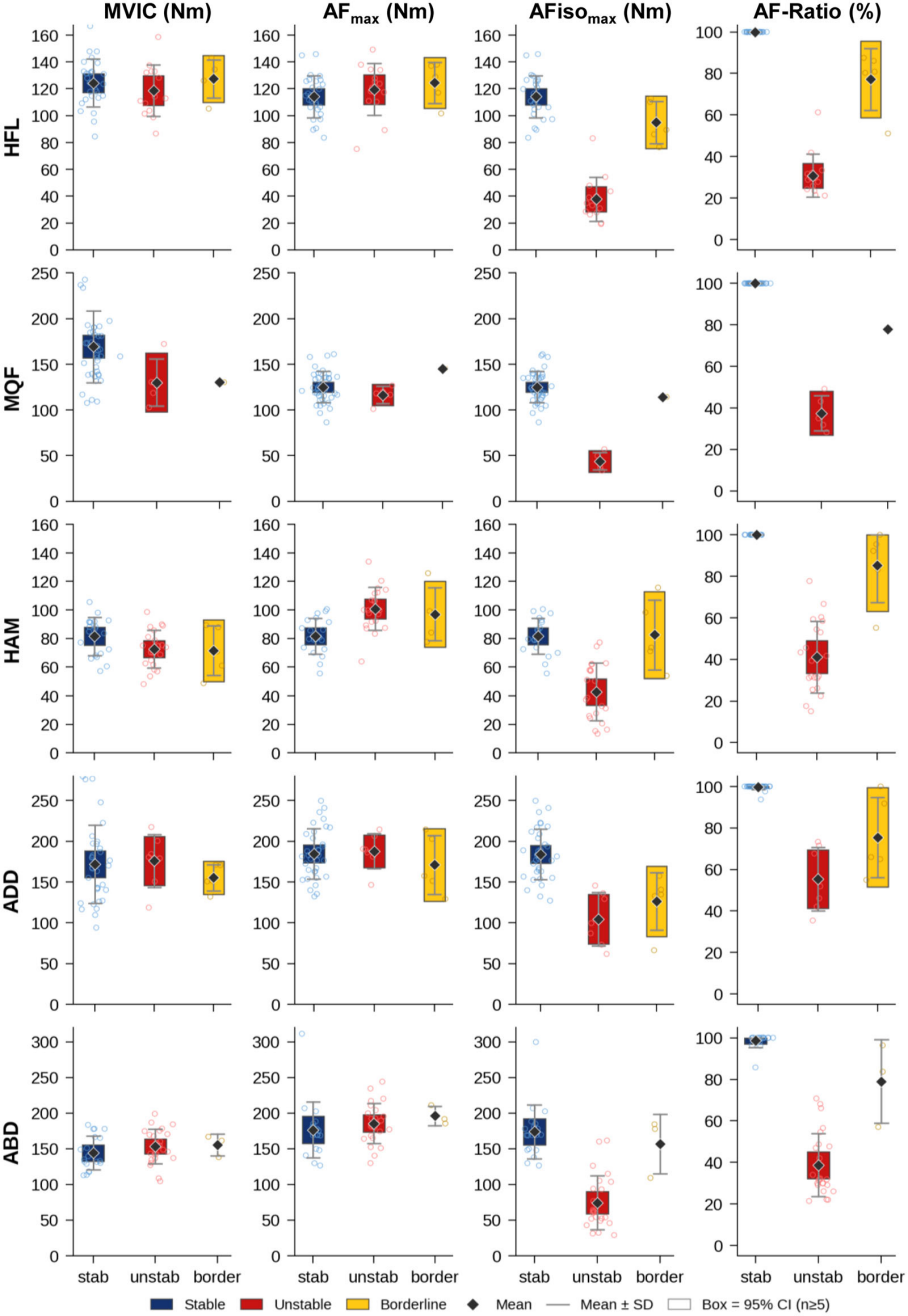
Force parameters by stability classification per muscle group (*n* = 230). Displayed are MVIC, AF_max_, AFiso_max_ (Nm) and AF‐Ratio (%) separated per muscle group (HFL: hip flexors, MQF: knee extensors, HAM: knee flexors, ADD: hip adductors, ABD: hip abductors) and stability classification stable (stab; blue), unstable (unstab; red) and borderline (border; gold). Box = 95% CI (omitted if *n* < 5, cut if AF‐Ratio > 100%); diamond = arithmetic mean, whiskers = M ± SD; circles = individual values. Sample sizes: HFL: stable 27, unstable 14, borderline 5; MQF: 40/5/1; HAM: 20/21/5; ADD: 34/7/5; ABD: 19/24/3. AFiso_max_, maximum isometric Adaptive Force; AF_max_, maximum AF; AF‐Ratio, AFiso_max_/AF_max_; CI, confidence interval; MVIC, maximum voluntary isometric contraction; SD, standard deviation.

Table 1 and Figure 4 show all force parameters per muscle group and stability category. Across all muscles, MVIC and AF_max_ did not differ significantly between stability categories, indicating that maximal force production was not impaired despite instability. At the individual muscle level, HAM showed an inverse pattern with higher AF_max_ in unstable muscles, reflecting the capacity to generate higher force during muscle lengthening despite instability.

**Table 1:** Arithmetic means, standard deviations and 95% CIs (*M* ± SD [95% CI]) of MVIC, AF_max_, AFiso_max_ and AF‐Ratio pooled and for each muscle group, separated by stability categories (stable, unstable, borderline).

Muscle	Category	*n*	MVIC (Nm)	AF_max_ (Nm)	AFiso_max_ (Nm)	AF‐Ratio (%)
Pooled	Stable	140	145.3 ± 46.3 [137.6, 153.1]	138.2 ± 43.8 [130.9, 145.5]	137.7 ± 43.3 [130.5, 145.0]	99.8 ± 1.4 [99.5, 100.0]
	Unstable	71	123.1 ± 42.4 [113.1, 133.2]	142.8 ± 44.2 [132.3, 153.2]	58.7 ± 34.7 [50.4, 66.9]	39.4 ± 15.6 [35.7, 43.1]
	Borderline	19	124.6 ± 37.4 [106.6, 142.6]	141.9 ± 42.5 [121.4, 162.3]	110.8 ± 37.0 [92.9, 128.6]	79.2 ± 16.2 [71.4, 87.0]
	*p*/*ω* ^2^ (all categories)		0.310/0.00	0.054/0.02	**<0.001/0.53**	**<0.001/0.88**
HFL	Stable	27	124.3 ± 17.8 [117.2, 131.4]	114.1 ± 15.6 [107.9, 120.3]	114.1 ± 15.6 [107.9, 120.3]	100.0 ± 0.0 [100.0, 100.0]
	Unstable	14	118.5 ± 19.2 [107.4, 129.6]	119.5 ± 19.2 [108.4, 130.6]	37.7 ± 16.5 [28.2, 47.3]	30.8 ± 10.3 [24.9, 36.8]
	Borderline	5	127.4 ± 14.1 [109.9, 144.9]	124.5 ± 15.3 [105.5, 143.5]	94.9 ± 15.8 [75.4, 114.5]	77.2 ± 14.9 [58.7, 95.7]
	*p*_adj_/*d* [95% CI]		1.000/0.32 [−0.33, 0.97]	1.000/−0.32 [−0.97, 0.33]	**<0.001/4.80** [3.56, 6.05]	**<0.001/11.62** [8.96, 14.28]
MQF	Stable	40	169.3 ± 39.4 [156.7, 181.9]	125.2 ± 17.0 [119.8, 130.6]	125.2 ± 17.0 [119.8, 130.6]	100.0 ± 0.0 [100.0, 100.0]
	Unstable	5	130.1 ± 25.8 [98.1, 162.2]	116.6 ± 9.5 [104.8, 128.3]	43.9 ± 9.6 [32.0, 55.8]	37.5 ± 8.6 [26.8, 48.1]
	Borderline	1	130.2	145.1	114.3	78.0
	*p*_adj_/*d* [95% CI]		0.108/1.02 [0.07, 1.98]	0.618/0.53 [−0.41, 1.46]	**<0.001/4.96** [3.56, 6.36]	**<0.001/23.93** [18.79, 29.07]
HAM	Stable	20	81.6 ± 13.4 [75.3, 87.9]	81.7 ± 12.5 [75.9, 87.5]	81.7 ± 12.5 [75.9, 87.5]	100.0 ± 0.0 [100.0, 100.0]
	Unstable	21	72.6 ± 13.2 [66.6, 78.6]	100.8 ± 15.1 [93.9, 107.6]	42.7 ± 20.1 [33.6, 51.8]	41.1 ± 17.1 [33.3, 48.9]
	Borderline	5	71.6 ± 17.4 [50.0, 93.2]	97.0 ± 18.5 [74.0, 120.0]	82.5 ± 24.4 [52.3, 112.8]	85.2 ± 17.9 [63.0, 107.4]
	*p*_adj_/*d* [95% CI]		0.184/0.68 [0.05, 1.31]	**<0.001/**−**1.37** [−2.06, −0.69]	**<0.001/2.32** [1.52, 3.12]	**<0.001/4.81** [3.58, 6.04]
ADD	Stable	34	171.9 ± 47.8 [155.2, 188.6]	184.5 ± 31.0 [173.7, 195.3]	184.1 ± 31.2 [173.2, 195.0]	99.7 ± 1.1 [99.3, 100.1]
	Unstable	7	175.9 ± 32.5 [145.8, 205.9]	187.9 ± 21.7 [167.8, 207.9]	104.7 ± 32.8 [74.4, 134.9]	55.4 ± 15.3 [41.2, 69.6]
	Borderline	5	155.2 ± 16.3 [135.0, 175.5]	171.0 ± 36.1 [126.1, 215.9]	126.3 ± 34.9 [82.9, 169.7]	75.5 ± 19.3 [51.5, 99.5]
	*p*_adj_/*d* [95% CI]		1.000/−0.09 [−0.90, 0.73]	1.000/−0.11 [−0.93, 0.70]	**0.002/2.52** [1.54, 3.51]	**0.001/7.27** [5.46, 9.08]
ABD	Stable	19	144.1 ± 23.8 [132.6, 155.6]	176.4 ± 39.2 [157.5, 195.3]	173.8 ± 37.8 [155.6, 192.0]	98.8 ± 3.4 [97.1, 100.4]
	Unstable	24	153.2 ± 24.2 [143.0, 163.5]	185.4 ± 28.2 [173.5, 197.3]	74.5 ± 37.6 [58.6, 90.4]	38.7 ± 15.1 [32.3, 45.1]
	Borderline	3	155.3 ± 15.3 [117.5, 193.2]	196.0 ± 13.4 [162.6, 229.3]	157.0 ± 41.6 [53.7, 260.3]	79.0 ± 20.1 [29.1, 129.0]
	*p*_adj_/*d* [95% CI]		1.000/−0.38 [−0.99, 0.23]	1.000/−0.27 [−0.87, 0.34]	**<0.001/2.64** [1.81, 3.47]	**<0.001/5.20** [3.92, 6.48]

*Note*: For pooled comparisons across all three stability categories, *p* values and effect sizes omega squared (*ω* ^2^) are given (Welch's ANOVA; for absolute force parameters calculated on *z*‐standardized values). For individual muscle groups, Bonferroni‐adjusted *p* values (*p*_adj_) and Cohen's *d* with 95% CIs for stable versus unstable comparisons are reported. Significant values are in bold.

Abbreviations: ABD, hip abductors; ADD, hip adductors; AFiso_max_, maximum isometric Adaptive Force; AF_max_, maximum AF; AF‐Ratio, AFiso_max_/AF_max_; ANOVA, analysis of variance; CIs, confidence intervals; HAM, hamstrings; HFL, hip flexors; MVIC, maximum voluntary isometric contraction; MQF, quadriceps femoris muscle; SD, standard deviation.

In contrast, AFiso_max_ was markedly lower in unstable versus stable, resulting in an average AF‐Ratio of 39% versus ~100%; where unstable HFL showed the strongest reduction (−69%) and ADD the lowest, still with an average reduction of −45%. Borderline values were intermediate between stable and unstable, supporting the validity of the three‐category classification as an ordinal scale.

The strongest discrimination between stability categories was found for AF‐Ratio, accounting for 88% of the variance (post hoc: stable–unstable: *d* = 6.62, 95% CI [5.92, 7.31]; stable–borderline: *d* = 3.64, [3.02, 4.27]; borderline–unstable: *d* = 2.52, [1.90, 3.15]; all *p *< 0.001). The single‐trial AF‐Ratio distributions by muscle and stability category (stable/unstable; algorithm‐ and examiner‐based) are illustrated in Supporting Information S1: Figure 2. AFiso_max_ similarly discriminated all three categories (post‐hoc: stable–unstable: *d* = 2.42, [2.05, 2.79]; borderline–unstable: *d* = 1.44, [0.90, 1.99]; both *p* < 0.001; stable–borderline: *d* = 0.69, [0.21, 1.18], *p* = 0.124).

While absolute force parameters (MVIC, AF_max_, AFiso_max_) differed substantially between muscles within each stability category (all *p* < 0.001, except for AFiso_max_ borderline: *p* = 0.108), reflecting inherent differences in force‐generating capacity, the AF‐Ratio was largely consistent across muscle groups. Within unstable muscles, a small but significant difference remained (*p* = 0.020), driven solely by higher AF‐Ratio values in ADD compared to HFL (*p*_adj_ = 0.042); all other pairwise comparisons were not significant. Within stable and borderline, the AF‐Ratio did not differ between muscles (*p* = 0.829 and *p* = 0.871). Thus, unlike the absolute force parameters, the AF‐Ratio did not differ systematically between muscle groups, suggesting it largely normalizes for muscle‐specific force differences.

The three absolute force parameters showed no pairwise differences in stable muscles after Bonferroni correction (*F*(1.0,139.9) = 4.78, *p* = 0.030, *η*_p_^2 ^= 0.03; all *p*_adj_ > 0.07), indicating equivalence of HIMA (AFiso_max_), PIMA (MVIC) and peak Adaptive Force (AF_max_); however, ABD showed a notably different pattern, with AFiso_max_ exceeding MVIC on average by ~21% (*d* = 0.92, [0.39, 1.46]). In unstable muscles, all parameters differed significantly (*F*(1.7,119.3) = 278.66, *p* < 0.001, *η*_p_^2 ^= 0.80; all *p*_adj_ < 0.001): AFiso_max_ was on average 52% lower than MVIC (*d* = −1.73, [−2.09, −1.36]) and 59% lower than AF_max_ (*d* = −2.92, [−3.45, −2.39]), while AF_max_ exceeded MVIC by 16% (*d* = 0.72, [0.46, 0.98]). This demonstrates that isometric holding capacity is selectively impaired, whereas pushing strength and peak eccentric force remain unaffected or even increased.

Correlations between absolute force parameters revealed a graded decoupling pattern. In stable muscles, they were moderately to strongly intercorrelated (AFiso_max_–AF_max_: *r* = 0.83; AFiso_max_–MVIC: *r* = 0.49; AF_max_–MVIC: *r* = 0.51; all *p* < 0.001). In unstable muscles, AF_max_–MVIC remained essentially unchanged (*r* = 0.40, *p* < 0.001; stable vs. unstable: Fisher's *z* = 0.89, *p* = 0.375), whereas the AFiso_max_–AF_max_ correlation was significantly reduced (*r* = 0.61, *p* < 0.001; *z* = 3.32, *p* < 0.001), and AFiso_max_–MVIC additionally no longer reached significance within the unstable group (*r* = 0.22, *p* = 0.069; *z* = 2.09, *p* = 0.036).

### Comparison with the conventional H:Q ratio

The H:Q ratio showed no difference between unstable versus stable HAM (49.8 ± 12.4% vs. 49.4 ± 9.7%; *p* = 0.913, *d* = −0.04, [−0.67, 0.59]; limb level, *n* = 46; based on examiners' rating); overall, 89% of limbs fell below the commonly used 60% H:Q threshold (average across all limbs: 48.2 ± 10.9%). In contrast, the AF‐Ratio discriminated strongly, both averaged across HAM and MQF (60.6 ± 14.1% vs. 98.6 ± 5.6%; *p* < 0.001, *d* = 3.64, [2.60, 4.68]) and for HAM alone (37.0 ± 14.2% vs. 99.6 ± 1.7%; *p* < 0.001, *d* = 6.43, [4.84, 8.03]). No correlation was observed between the H:Q ratio and the AF‐Ratio of HAM at limb level (Spearman *r* = 0.13, *p* = 0.408).

## DISCUSSION

This cross‐sectional study assessed muscle stability by AF in semi‐professional football players. The main findings were the high prevalence of stability deficits prior to the season, and the selective impairment of the holding capacity in unstable muscles—reduced on average by over 60% compared to maximal force output—while maximal pushing strength and peak eccentric force remained unaffected. These findings suggest that reduced holding capacity may be common in players fully participating in training, and that muscle instability represents a selective impairment of adaptive holding capacity that is not captured by traditional strength assessment. Given the well‐documented limitations of strength screening in predicting injuries [3, 18, 19, 21, 39], the assessment of holding capacity offers a complementary framework, capturing a distinct neuromuscular construct which is theoretically relevant to injury‐prone movements.

### Prevalence and muscle‐specific patterns

Stability deficits were highly prevalent (83% of players, 74% of limbs, 39% of all tested muscle groups), with ABD (52%) and HAM (46%) showing the highest instability rates and MQF the lowest (11%). The high prevalence should be interpreted in the specific testing context: an intensive preseason preparation period with high cumulative training loads in a single semi‐professional team characterized by specific training and recovery practices. The observed prevalence may therefore not directly transfer to football players in other competitive settings or at different points in the season; replication in independent cohorts is warranted. The association of stability deficits with musculoskeletal complaints, including load‐related/generalized complaints, will be addressed in a companion paper.

The particularly high instability rate of ABD (52%) is noteworthy in light of their functional role in football. Video analyses of non‐contact ACL injuries consistently show hip abduction at initial contact and dynamic knee valgus during the injury event [14], precisely the loading conditions under which hip abductors must provide isometric and eccentric control. Previous findings on hip abduction strength and injury risk are inconsistent [4, 22, 24, 38], which may reflect a mismatch between the assessed function (pushing strength) and the functionally relevant demand on hip abductors (adaptive holding). AFiso_max_ exceeded MVIC by 22% in stable ABD—a pattern not observed in other muscle groups—suggesting that for these stabilizers, adapting to external loads is the primary habitual demand while the pushing task may represent an unfamiliar activation pattern potentially underestimating the muscle's true force capacity. Given their role in controlling frontal plane pelvic alignment during football‐specific movements [14], reduced ABD holding capacity during landing may contribute to uncontrolled knee valgus moments, a key mechanism in non‐contact ACL injuries [14].

The high rate of HAM instability aligns with epidemiological data identifying hamstrings as the most frequently injured muscle in football [15], yet none of 13 strength‐related variables reached significance in a meta‐analysis of 78 prospective studies [19]. The high prevalence of HAM stability deficits may offer an explanation, as holding capacity is not captured by conventional strength testing. Moreover, the sensitivity of holding capacity to proprioceptive irritation [7, 8] and nociception [30] may open a new perspective on the elevated risk of re‐injury following previous hamstring strain and ACL reconstruction [19], where the semitendinosus is commonly harvested as graft material; tissue damage and subsequent remodelling may further compromise HAM stability. This possibility warrants investigation.

The relatively low instability rate for ADD (15%, stability deficits including borderline: 26%) contrasts with adductors being the second most frequently injured muscle in football [15]. The mechanisms underlying this discrepancy remain speculative. One possibility is that adductor loading in sport‐specific situations depends on dynamic pelvic stability provided by the hip abductors; with impaired ABD holding capacity, adductor strain may increase under conditions not replicated in the non‐weight‐bearing supine testing position. Alternatively, given the functional synergy between adductors and the medial hamstring group, adductors may be overloaded when compensating for HAM instability—a mechanism that would likewise not be captured in isolated supine testing. Both explanations remain hypothetical and warrant investigation in dynamic weight‐bearing conditions.

The low instability rate of MQF (11%) may partly reflect insufficient examiner force capacity for the strong knee extensors in some players. However, unstable MQF muscles began yielding at ~38% of their peak force, whereas examiners reached twice that value in stable muscles, indicating the force level was well within the applied range. The low rate therefore appears to be a genuine finding rather than a methodological artefact. The present findings suggest that multi‐joint muscles crossing the knee—such as HAM and HFL—as well as proximal stabilizers (ABD) may be more relevant from a stability perspective, although typically less emphasized in conventional screening. Notably, the rectus femoris component of MQF—tested as part of the HFL in its hip flexion function—showed a considerably higher stability deficit rate (41%, including borderline) than MQF in its knee extension function (13%), suggesting that biarticular function may be more susceptible to stability deficits.

### Dissociation between holding capacity and pushing strength

A key finding was the selective and clear impairment of holding capacity in unstable muscles, while MVIC and AF_max_ remained unaffected and all force parameters were equivalent in stable muscles—demonstrating that instability is not a deficit in force production capacity per se. This is further supported by similar rates of force development across stability categories (*p* = 0.514), confirming that the observed deficits reflect differences in holding capacity rather than in force application. The dissociation is in line with the proposed differentiation between HIMA and PIMA, suggested to involve distinct motor control strategies [25, 28, 29]. The AF assessment challenges the neuromuscular system beyond a simple HIMA with constant load, as the participant must adapt to a progressively rising external load, demanding continuous proprioceptive feedback and rapid motor adjustments [6, 34]—an adaptive neuromuscular function highly relevant to sport‐specific motor demands. In contrast, PIMA simply requires maximizing force output against a fixed resistance, which does not necessitate such complex regulation.

The more complex control requirements of adaptive holding capacity may explain its high vulnerability, as shown in previous studies by an instant reduction upon presentation of impairing stimuli [7, 8, 31, 34, 35, 37]—suggesting the involvement of central regulatory circuits shared between motor control and the processing of these inputs [6, 34, 37]. Traditional strength testing relying on PIMA may be fundamentally unable to detect such deficits. For football players, this implies that neuromuscular protection of joint structures may be compromised when these central circuits are affected—even when maximal strength is fully preserved—a mechanism that could plausibly contribute to injury vulnerability. Notably, the Nordic hamstring exercise—whose preventive effect remains debated [1, 9, 11, 20, 42]—shares biomechanical similarities with adaptive holding, and cases of limited effectiveness may partly reflect unresolved underlying causes for instability not addressed by exercise alone.

All three force parameters depend on both motor capacity (capacitive component) and central control (reactive component), but probe different control aspects: MVIC tests sustained activation against fixed resistance, AF_max_ adaptive regulation under rising load without isometric restriction, and AFiso_max_ additionally requires maintaining the isometric state—thus most specifically testing adaptive holding control. In stable muscles, all three pairs were significantly correlated, with the strongest association for AFiso_max_–AF_max_ (*r* = 0.83) and comparable AFiso_max_–MVIC (*r* = 0.49) and AF_max_–MVIC (*r* = 0.51) correlations, indicating that intact adaptive holding control allows AFiso_max_ to reach the maximum force capacity, while both AF parameters share the capacitive component captured by MVIC. In unstable muscles, a graded pattern of decoupling emerged: AF_max_–MVIC remained comparable (*r* = 0.40; Fisher's z = 0.89, *p* = 0.375), whereas both AFiso_max_ correlations decreased significantly (AFiso_max_–MVIC: *r* = 0.22, z = 2.09, *p* = 0.036; AFiso_max_–AF_max_: *r* = 0.61, z = 3.32, *p* < 0.001). The latter is particularly informative as both parameters are extracted from the same trial, isolating the adaptive holding component as the selectively affected dimension. Together, these findings position AF_max_ as sharing the capacitive motor component with MVIC, while AFiso_max_ additionally captures the adaptive control component, which fluctuates with the immediate sensorimotor context independently of the underlying motor capacity. A single AF trial may thus characterize both components. The self‐normalized AF‐Ratio isolates the regulatory quality of holding under load—a metric responsive to current neuromuscular state, in contrast to the comparatively stable motor capacity. AF is therefore best understood as a control‐determined parameter: motor capacity sets the upper boundary, but adaptive holding control determines to what extent this capacity is actually expressed under load. The empirical signature of this relationship is the selective decoupling of AFiso_max_ from MVIC in unstable muscles, while MVIC itself remains unaffected—demonstrating that the deficit resides in the regulatory domain, not in the available force.

### Comparison of AF‐Ratio to the conventional H:Q ratio

The widely assessed H:Q ratio has shown limited predictive validity for injuries [5, 12, 19, 21, 39]. The present findings align with this, as the H:Q ratio failed to discriminate between stable and unstable HAM (*d* = −0.04). The majority of limbs (89%) fell below the conventional 60% cutoff [5, 12]—potentially reflecting differences in population, training level or methodology (see Limitations). In contrast, the AF‐Ratio discriminated strongly, both averaged across HAM and MQF (*d* = 3.64) and for HAM alone (*d* = 6.43). The two parameters were not correlated, suggesting they capture fundamentally different aspects of neuromuscular function: whereas the H:Q ratio reflects inter‐muscular strength balance, the AF‐Ratio captures within‐muscle neuromuscular control quality, the latter being more directly relevant to the stabilization demands of injury‐prone movements. Moreover, the AF‐Ratio is derived from a single muscle—normalizing the holding capacity to peak force—whereas the H:Q ratio requires separate maximal tests of two muscle groups.

Although the reconsideration of isokinetic strength screening has been recommended [18, 23], the response has largely been to monitor the same parameters more frequently rather than questioning whether maximal force production is the appropriate construct to assess [19, 21]. The present findings suggest that the construct itself may need to be reconsidered.

### AF‐Ratio: A muscle‐independent stability parameter and potential implications

The AF‐Ratio emerged as the strongest discriminator between stability categories, accounting for 88% of the variance with very large effect sizes (Cohen's *d* up to 23.9). These reflect the qualitative nature of the underlying distinction. Stable and unstable behaviour are not gradations on a continuous scale of strength but represent two distinct motor states: maintaining isometric position versus failure to adapt with transition to eccentric muscle action under increasing load. The AF‐Ratio captures this categorical transition through continuous quantification, which mechanistically produces effect sizes exceeding those typical of strength comparisons within a single motor state. Although uncommon in biological systems, the magnitude of effect sizes is supported by the robustness across alternative thresholds and classification criteria (Supporting Information S1: Sections 1.2 and 2.1), the even stronger discrimination under examiners' clinical classification and the contrast with the conventional H:Q ratio in the same sample. This pattern most likely reflects a genuine biological dichotomy between two qualitatively distinct motor states rather than an artefact of construct definition or scaling.

In contrast to absolute force parameters (MVIC, AF_max_, AFiso_max_), where between‐muscle differences emerged, the AF‐Ratio did not differ significantly between muscle groups within stable or borderline categories (both *p* > 0.820). Within unstable muscles, a small but significant between‐muscle difference remained (*p* = 0.020), driven solely by higher AF‐Ratio values in ADD compared to HFL (55% vs. 31%), both still showing a substantial reduction. This remaining difference could reflect the distinct biomechanical characteristics of the ADD test—where angular displacements during yielding are smaller due to the test geometry—or genuine muscle‐specific differences.

Nevertheless, the results suggest that the AF‐Ratio may serve as a universal stability parameter. Previous research supports that the holding capacity discriminates between stable and unstable muscles and that the AF‐Ratio is independent of factors such as assessed muscle, participants' sex, examiner and experimental approach [30]. Being self‐referenced to each muscle's own peak force, it eliminates the need for external reference values and effectively normalizes for muscle‐specific force differences, enabling direct comparison of stability across different muscle groups—a desirable property for a clinical assessment tool. However, its broad sensitivity across different modulatory influences [7, 8, 30, 31, 32, 34, 37] underscores the AF‐Ratio as a marker of general neuromuscular integrity and implies limited specificity: A reduction indicates impaired holding capacity but does not, by itself, reveal the underlying cause.

The characteristics of AF render it particularly relevant to injuries, as it addresses neuromuscular function that is challenged in injury‐prone motions involving stabilization and active deceleration [2, 14, 16, 26]—functions not captured by traditionally assessed pushing strength. In common football situations (e.g., direction changes, landings with rotation/valgus), the muscles surrounding the knee and hip must stabilize the joints while adapting to the increasing external forces—precisely the function assessed by AF. With unstable muscles that begin to lengthen at less than 50% of their maximal capacity (as observed here), premature yielding may subject passive structures to loads they are not designed to absorb. Consequently, it is hypothesized that muscle instability, as defined by AF, could be a key risk factor for non‐contact injuries and the development of musculoskeletal complaints, as the function of stabilization under load is likely impaired; with full muscle stability, muscles can stabilize up to their maximum force level, thereby protecting passive structures. This hypothesis requires verification in prospective studies.

The concept of muscle stability—distinct from muscle strength—offers a novel mechanistic perspective on the development of musculoskeletal complaints and non‐contact injuries and opens avenues for practical applications. Notably, holding capacity is acutely responsive to impairing and supportive stimuli [7, 8, 31, 34, 35, 37], underscoring its central neuromuscular control with potential for clinical assessment and targeted individualized interventions [32]; systematic investigation is warranted.

### Limitations

The sample was limited to male semi‐professional players from a single team, restricting generalizability to other populations, competitive levels or female athletes. The measurements were conducted during an intensive preseason preparation period, which may have transiently influenced the prevalence of stability deficits.

The classification boundaries and the AFiso_max_ detection threshold were derived from the present dataset; while sensitivity analyses confirmed robustness of the AF‐Ratio group differences across boundary and threshold variants (Supporting Information S1: Sections 1.2 and 2.1), generalizability requires verification on independent datasets. Formal intra‐ and inter‐rater reliability of the examiners' clinical ratings has not yet been quantified; reproducibility of the force profile within and between the two examiners has been demonstrated [6]. The aggregated classification based on three individual trials may mask isolated stability deficits, potentially diluting clear findings into borderline classifications; unlike random error in continuous measures, however, such a deviating trial may itself reflect genuine intermittent instability rather than noise. For hip adductors and abductors, the long lever arm spanning two joints (hip and knee) and the smaller test range of motion reduce the contrast between yielding and physiological background variability. This makes the boundary between stable and yielding behaviour less distinct than for the other muscles tested (Supporting Information S1: Section 2).

The H:Q ratio was calculated from MVIC tests with HAM and MQF measured in different positions (prone vs. supine), unlike standard isokinetic protocols. This limits comparison with normative isokinetic data; however, between‐group comparisons remain valid as all participants were measured identically.

## CONCLUSION

Stability deficits assessed by AF were highly prevalent in semi‐professional football players, with a selective and clear impairment of holding capacity in unstable muscles—with preserved maximal pushing strength—demonstrating that traditional strength testing may be unable to detect such deficits. These findings suggest that the adaptive holding capacity captures a distinct neuromuscular construct beyond conventional strength assessment, offering a novel mechanistic framework for understanding the development of musculoskeletal complaints and non‐contact injuries. Further investigations in larger and more diverse samples, evaluation of the association between stability deficits and musculoskeletal complaints, and prospective studies on the relationship with injuries are required to assess the potential utility of stability assessment in clinical practice.

## AUTHOR CONTRIBUTIONS

All authors contributed to the study conception and design. Data collection was performed by Laura V. Schaefer and Frank N. Bittmann. Data analysis was performed by Laura V. Schaefer and Jaali Ulrich. The first draft of the manuscript was written by Laura V. Schaefer, and all authors commented on previous versions of the manuscript. All authors read and approved the final manuscript.

## CONFLICT OF INTEREST STATEMENT

The authors declare no conflicts of interest.

## ETHICS STATEMENT

The study was conducted in accordance with the Declaration of Helsinki and approved by the Ethics Committee of the University of Potsdam (protocol number: 35/2018; 17 October 2018). Informed consent was obtained from all individual participants included in the study. The authors affirm that human research participants provided informed consent for publication of the images in Figure 1a–e.

## Supporting information

Supporting File 1.

## Data Availability

The data that support the findings of this study are available from the corresponding author upon reasonable request.
