# Supplementary material for: High prevalence of muscle stability deficits in semi‐professional football players—Adaptive Force assessment reveals selective impairment of holding capacity with preserved maximal strength: A cross‐sectional study
Source: Knee Surg Sports Traumatol Arthrosc. 2026 Jul 7;34(8):2987–3001. doi: 10.1002/ksa.70525 (PMC13418286; doi:10.1002/ksa.70525)
Supplement: Supplementary file 1 — Supporting File 1. [file KSA-34-2987-s001.pdf]

JEO
The official open access journal of ESSKA

KSSTA
The clinical journal of ESSKA

– Supporting Information –

High prevalence of muscle stability deficits in semi-professional football players—Adaptive Force assessment reveals selective impairment of holding capacity with preserved maximal strength: A cross-sectional study

Schaefer LV, Bittmann FN, Ulrich J, Prill R, Becker R

Section 1: Stability classification 2
SI Table 1. Classification boundaries. 3
Section 1.1 Validation: examiner vs. algorithm-based classification 3
SI Table 2. Examiner vs. algorithm-based classification. 4
Section 1.2 Sensitivity analysis across classification boundaries 4
SI Table 3. Classification boundaries for sensitivity analysis. 4
SI Figure 1. Sensitivity analysis of AF-Ratio group differences across classification methods and boundaries. 5
Section 2: Thresholds for detection of $AFiso_{max}$ 6
Section 2.1 Robustness analyses of $AFiso_{max}$ detection 7
SI Table 4. Overall concordance per threshold set. 8
SI Table 5. Per-muscle concordance for reference thresholds. 8
SI Table 6. AF-Ratio discrimination across thresholds and classification criteria. 9
SI Figure 2. Distribution of individual-trial AF-Ratio values by muscle and stability category. 9

Section 1: Stability classification

Automated (algorithm-based) stability classification was based on the yielding parameter c · AFmax (°/s), which quantifies the overall degree of muscular yielding. Muscle-specific boundaries were applied to classify each trial as stable, borderline, or unstable (SI Table 1). For hip flexors (HFL), knee extensors (MQF), and knee flexors (HAM), classification was based solely on the yielding parameter $c \cdot AF_{max}$. For hip adductors (ADD) and hip abductors (ABD), which exhibit smaller gyroscope amplitudes in the borderline range, supplementary Euler angle–derived parameters were used to resolve ambiguous cases. These supplementary parameters were used exclusively for classification, not for the determination of $AFiso_{max}$. The classification and $AFiso_{max}$ detection are both derived from the least-squares fit coefficient c, but use different approaches and cut-offs: the classification employs muscle-specific boundaries of $c \cdot AF_{max}$ to distinguish stable, borderline and unstable behaviour, whereas the $AFiso_{max}$ detection identifies the approximate onset of yielding (see Section 2).

Three measurements per muscle and side were aggregated into a single classification for statistics: three identical ratings yielded that category; mixed stable and unstable ratings (without borderline) were classified as borderline; where borderline ratings were present, the majority category was assigned.

Agreement between the manual and automated classification was assessed at two levels: individual measurements (n = 685) and aggregated muscle–side combinations (n = 230). Cohen's kappa (κ) was calculated for the three-category agreement, both unweighted and quadratically weighted to account for the ordinal nature of the classification (stable < borderline < unstable). For binary classification (stable vs. unstable), sensitivity, specificity, and accuracy were determined using the examiner's assessment as reference, excluding examiner-borderline cases (which lack a definitive reference). Kappa values were interpreted according to Landis and Koch.[1]

[1] Landis JR, Koch GG. The Measurement of Observer Agreement for Categorical Data. *Biometrics*. 1977;33(1):159. https://doi.org/10.2307/2529310.

SI Table 1. Classification boundaries.

	yielding parameter c · AFmax (°/s)				Euler-Parameters (°)
**Muscle**	**Stable**	**Unstable**	**Borderline**		
HFL	< 4	> 6	4 – 6		—
MQF	< 3	> 6	3 – 6		—
HAM	< 3	> 5	3 – 5		—
ADD	< 1	> 3	1 – 3	1 – 3	stable: MM5020 < 6<br>unstable: MM5020 >8 AND MM9090 > 1<br>else: borderline
ABD	< 1	> 3.5	1 – 3.5	1 – 2.5	stable: MM8020 <5<br>else: borderline
				2.5 – 3.5	unstable: MM8020 ≥6<br>else: borderline

MM = maximum monotonic (i.e., uninterrupted, without directional reversal) angular displacement in the yielding direction extracted from Euler angle signals within defined force ranges:
MM5020 covers 50–20% AF_{max} (broad range including the full yielding phase)
MM8020 covers 80–20% AF_{max}
MM9090 covers 90–90% AF_{max} (high-force region only)

Section 1.1 Validation: examiner vs. algorithm-based classification

At the individual measurement level (n = 685), overall three-category agreement was 94.2% (645/685; κ = 0.89; quadratically weighted κ = 0.97; SI Table 2). Among the 627 trials with a definitive examiner classification (stable or unstable), accuracy was 98.7% (619/627), with no stable–unstable misclassification (i.e., no examiner-stable cases classified as unstable by the algorithm and vice versa). The eight discrepancies (1.3%) all involved the borderline category (algorithm assigned borderline classification to trials rated as stable (n = 6) or unstable (n = 2) by the examiner).

Of the 58 measurements rated as borderline by the examiner, the algorithm classified 26 (44.8%) as borderline, 12 (20.7%) as stable, and 20 (34.5%) as unstable. This distribution is consistent with the nature of borderline cases as transitional states, where a quantitative boundary-based algorithm resolves ambiguity that the manual assessment left open. Importantly, no measurement rated as stable by the examiner was classified as unstable by the algorithm, or vice versa.

At the aggregated level (n = 230), agreement was 93.5% (215/230; κ = 0.88; weighted κ = 0.96). Among the 202 cases with a definitive examiner classification, accuracy was 98.5% (199/202), again with no stable–unstable misclassifications. Agreement per muscle group ranged from 87.0% (HAM) to 97.8% (MQF). Based on the high agreement between manual and automated

classification, the algorithm-based classification was chosen as the primary criterion in all analyses, as it provides an objective, reproducible, and rater-independent categorization; examiners' ratings were retained as an independent validation criterion.

SI Table 2. Examiner vs. algorithm-based classification.
Number of single trials per stability category.

Examiner / Algorithm	Stable	Borderline	Unstable	Sum
**Stable**	420	12	0	**432**
**Borderline**	6	26	2	**34**
**Unstable**	0	20	199	**219**
**Sum**	**426**	**58**	**201**	**685**

Section 1.2 Sensitivity analysis across classification boundaries

To assess the robustness of the AF-Ratio group differences against the specific classification boundaries used in the main analysis, the algorithm-based classification was repeated with four boundary sets: the *reference* boundaries used throughout the study (Table 1), *strict* boundaries (lowered for stable / raised for unstable; broader borderline range), *liberal* boundaries (raised for stable / lowered for unstable; minimized borderline range), and *shifted* boundaries (both lowered while keeping the borderline range constant; Table 3). The supplementary Euler angle–derived parameters for ADD and ABD (Table 1) remained unchanged.

The classifications were aggregated to muscle level using the rules described above. The AF-Ratio was compared between stable and unstable muscle groups using Cohen's *d* with 95% confidence intervals ($AFiso_{max}$ detection threshold was kept unchanged across all variants, isolating the effect of the classification boundaries from the $AFiso_{max}$ detection itself). The examiner-based classification was included as a methodologically independent reference.

SI Table 3. Classification boundaries for sensitivity analysis.
The classification boundaries for four different variants are given for stable and unstable (stable │ unstable) category separated by each muscle. Reference: used for main analysis in the present study; strict: lowered boundary for stable/raised boundary for unstable (broader borderline range), liberal: raised for stable/lowered for unstable (minimized borderline range), shifted: both boundaries lowered (borderline range constant).

	HFL	MQF	HAM	ADD	ABD
Reference	< 4 │ > 6	< 3 │ > 6	< 3 │ > 5	< 1 │ > 3	< 1 │ > 3.5
Strict	< 3 │ > 7	< 2 │ > 7	< 2 │ > 6	< 0.5 │ > 3.5	< 0.5 │ > 4
Liberal	< 4.5 │ > 5.5	< 3.5 │ > 5.5	< 3.5 │ > 4.5	< 1.25 │ > 2.75	< 1.25 │ > 3.25
Shifted	< 3 │ > 5	< 2 │ > 5	< 2 │ > 4	< 0.5 │ > 2.5	< 0.5 │ > 3

Across all four algorithmic boundary sets, the AF-Ratio discriminated strongly between stable and unstable muscle groups, with Cohen's *d* ranging from 5.77 [5.16, 6.39] (shifted) to 6.90 [6.18, 7.62] (strict); the reference boundaries yielded $d = 6.62$ [5.93, 7.31]. The examiner-based classification produced an even larger effect ($d = 8.02$ [7.19, 8.86]). All comparisons reached $p < 0.001$ (SI Figure 1). Sample sizes varied only marginally across the algorithmic variants (n_stable: 136–141; n_unstable: 70–76; n_borderline: 18–21).

Concordance between each algorithmic boundary set and the examiner classification remained high throughout (overall agreement 90.4–94.2% at trial level, 91.3–93.5% at muscle level; Cohen's κ = 0.82–0.89, indicating almost perfect agreement[1]). Accuracy among trials with a definitive examiner classification (stable/unstable) ranged from 95.5% to 98.9%. No direct stable–unstable misclassification occurred at the muscle level in any variant; at the trial level, such misclassifications were rare (0–4 of 685) and occurred exclusively in the direction

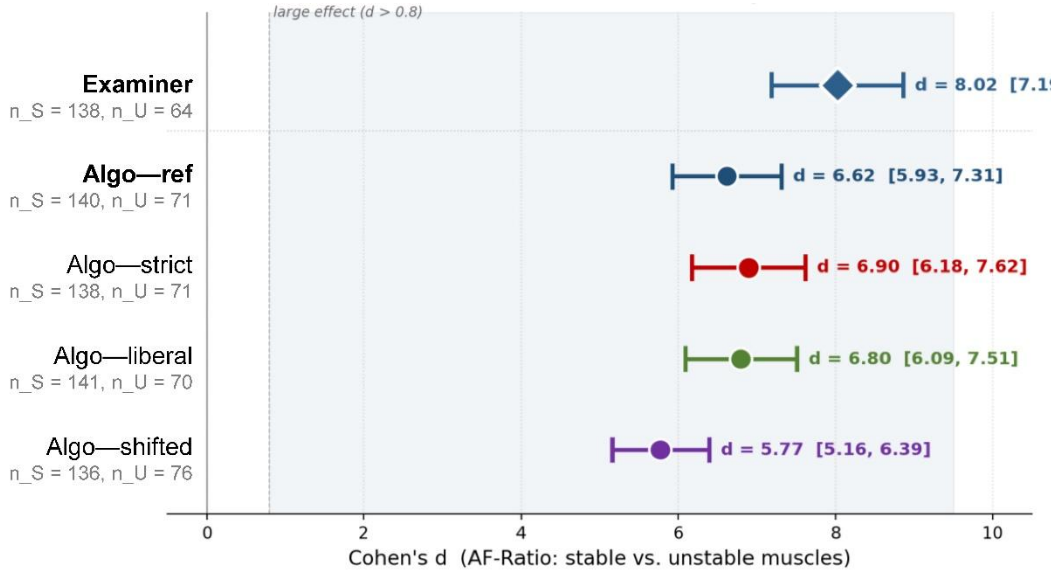

SI Figure 1. Sensitivity analysis of AF-Ratio group differences across classification methods and boundaries.

The forest plot illustrates the effect sizes (Cohen's d with 95% confidence intervals) comparing AF-Ratio between stable and unstable muscles regarding the following different classification approaches: 'Examiner' (based on examiner's tactile-kinaesthetic rating during AF assessment) and 'Algo' (algorithm-based on yielding coefficient c · AFmax (°/s), where c is the coefficient of the least-squares linear fit without intercept (gX = c · F) of angular velocity (gX) and force (F) signals of AF trials). For algorithm-based classification, different boundaries for yielding coefficient were considered: ref = used for main analysis in the study (see SI Table 1 for muscle-specific boundaries), strict = lowered boundaries for stable/raised boundaries for unstable (broader borderline range), liberal = raised for stable/lowered for unstable (minimized borderline range), shifted = both boundaries lowered (borderline range constant). Concrete muscle-specific boundaries are provided in SI Table 3. n_S = number of stable muscles, n_U = number of unstable muscles.

examiner-stable → algorithm-unstable, consistent with the mechanical expectation when boundaries are tightened. All remaining discrepancies involved transitions to or from the borderline category.

Together with the $AFiso_{max}$ sensitivity analysis (Section 2.1), these results demonstrate that the AF-Ratio group differences are robust to both the $AFiso_{max}$ detection threshold and the classification boundaries, and that the same pattern is obtained when the rater-based classification is used as grouping criterion. The discrimination of stable and unstable muscles by the AF-Ratio thus reflects a stable property of the construct rather than an artefact of the specific algorithmic parameters chosen.

Section 2: Thresholds for detection of $AFiso_{max}$

$AFiso_{max}$ was determined as the force at which c · F(t) first exceeded a defined detection threshold during force increase. At the applied thresholds (4 °/s: HFL, MQF, HAM; 2 °/s: ADD, ABD), the resulting angular displacement is well within the range commonly still considered quasi-isometric. Lower thresholds were applied for ADD and ABD to account for the smaller test range of motion in the respective settings.

The thresholds were calibrated such that the resulting AF-Ratio reflects the expected outcome based on prior research[2]: AF-Ratio close to 100% in clinically stable trials (where $AFiso_{max} \approx AF_{max}$; some tolerance for naturally occurring small oscillations and spring-like muscle behaviour, with AF-Ratio ≥ 95% considered consistent with stability) and substantially reduced in clinically unstable trials, where the muscle yields at submaximal intensities (AF-Ratio < 90%; cf. Schaefer & Bittmann (2025)[2], who reported AF-Ratio of ~56% (range: 0.1–0.91) in unstable trials).

Validation at individual trial level (n = 627 trials with stable or unstable rating) showed that the chosen muscle-specific thresholds achieved 98.7% overall concordance with this expectation (419/426 = 98.4% of stable trials at AF-Ratio ≥ 95%; 200/201 = 99.5% of unstable trials at AF-Ratio < 90%; SI Table 4). For HFL, MQF, and HAM, concordance was 100% in both directions (SI

[2] Schaefer LV, Bittmann FN. From strength to stability: muscular holding capacity characterized by Adaptive Force—relevance and implications of an alternative approach to musculoskeletal complaints and non-contact injuries. bioRxiv 2025.09.08.670731 [preprint]. https://doi.org/10.1101/2025.09.08.670731

Table 5). The lower concordance for ADD and ABD likely reflects the test geometry of these muscle groups: the long lever arm spans two joints (hip and knee), and the smaller overall test range of motion reduces the contrast between yielding and physiological background variability (subtle device tilting, oscillations, natural joint play of bi-articular muscle tests). This makes the boundary between stable and yielding behaviour less distinct than for the other muscles tested.

Section 2.1 Robustness analyses of AFiso$_{max}$ detection

To evaluate the robustness of the central findings, the AFiso$_{max}$ detection was repeated with five threshold sets: the reference muscle-specific thresholds (4 °/s for HFL, MQF, HAM and 2 °/s for ADD, ABD), stricter (3 and 1.5 °/s) and more liberal (5 and 2.5 °/s) muscle-specific alternatives, and two uniform thresholds (2 °/s and 3 °/s across all muscles). The resulting AF-Ratio was tested against two independent classification criteria: (i) examiners' clinical ratings, based on direct tactile perception of yielding during the test, serving as the clinical reference; and (ii) the algorithm-based classification used in the main analysis (Section 1).

The reference muscle-specific thresholds (4 and 2 °/s) achieved the highest combined concordance with the expected pattern based on prior research[2] of high AF-Ratio (≥ 95%) in clinically rated stable and substantially reduced values (< 90%) in unstable trials (98.7%, n = 627 individual trials; SI Table 4), with 100% concordance for HFL, MQF, and HAM, and > 95% for ADD and ABD (SI Table 5). The lower concordance for ADD and ABD likely reflects test-geometry-related variability (long lever arm spanning hip and knee, smaller test range of motion; see also the Limitations section in the main paper). Uniform thresholds showed lower overall concordance because they did not account for the test-geometry-related differences in angular displacement between muscle groups, justifying the use of muscle-specific thresholds.

Across all 10 combinations (5 thresholds × 2 classification criteria), the AF-Ratio discriminated strongly between stable and unstable muscle groups (Cohen's d = 4.30–10.01; all $p < 0.001$; SI Table 6). Examiner-based discrimination was consistently higher than algorithm-based (range of d: 4.80–10.01 vs. 4.30–9.00), reflecting the clear clinical distinction made by examiners; this also supports the choice of the algorithmic classification as the more conservative but rater-independent primary criterion in the main analysis (see Section 1). The single-trial AF-Ratio

distributions show clear separation between stable and unstable trials for HFL, MQF and HAM, with limited overlap restricted to ADD under the algorithm and to ADD and ABD under the examiners (SI Figure 2). Even the lowest discrimination across all tested combinations (Cohen's d = 4.30; uniform 3 °/s) substantially exceeds the threshold for a 'very large' effect, confirming the strength of the underlying construct. The consistency across all combinations demonstrates that the AF-Ratio captures the construct of muscle stability robustly—independently of the specific algorithmic parameters chosen—and objectively quantifies the clinical distinction made by the examiners. Replication on independent datasets, applying the present algorithm without re-calibration and comparing it against independent clinical ratings, is required to verify the generalizability of the approach.

SI Table 4. Overall concordance per threshold set.

Concordance of algorithmic AF-Ratio with examiners' classification at individual trial level (n = 627; stable or unstable ratings), using asymmetric cut-offs (stable: AF-Ratio ≥ 95%; unstable: AF-Ratio < 90%).

Threshold for AFisomax detection	Stable: AF-Ratio ≥ 95%	Unstable: AF-Ratio < 90%	Combined concordance
**Reference (4 and 2 °/s; muscle-specific)**	**419/426 (98.4%)**	**200/201 (99.5%)**	**98.7%**
Stricter (3 and 1.5 °/s; muscle-specific)	403/426 (94.6%)	200/201 (99.5%)	96.2%
More liberal (5 and 2.5 °/s; muscle-specific)	424/426 (99.5%)	190/201 (94.5%)	97.9%
Uniform 2 °/s (across all muscles)	401/426 (94.1%)	200/201 (99.5%)	95.9%
Uniform 3 °/s (across all muscles)	419/426 (98.4%)	185/201 (92.0%)	96.3%

The reference thresholds (4 and 2 °/s) provided the highest balanced concordance with examiners' classifications.

SI Table 5. Per-muscle concordance for reference thresholds.

Per-muscle concordance with examiners' classification at the reference muscle-specific thresholds (4 and 2 °/s).

Muscle	Stable: AF-Ratio ≥ 95%	Unstable: AF-Ratio < 90%	Combined concordance
**HFL**	85/85 (100.0%)	43/43 (100.0%)	**128/128 (100.0%)**
**MQF**	120/120 (100.0%)	12/12 (100.0%)	**132/132 (100.0%)**
**HAM**	65/65 (100.0%)	52/52 (100.0%)	**117/117 (100.0%)**
**ADD**	99/101 (98.0%)	25/26 (96.2%)	**124/127 (97.6%)**
**ABD**	50/55 (90.9%)	68/68 (100.0%)	**118/123 (95.9%)**
**Total**	**419/426 (98.4%)**	**200/201 (99.5%)**	**619/627 (98.7%)**

HFL, MQF, and HAM showed perfect concordance (100%). The lower concordance for ADD and ABD is discussed in the text.

SI Table 6. AF-Ratio discrimination across thresholds and classification criteria.

AF-Ratio discrimination between stable and unstable muscle groups across five thresholds for AFiso$_{max}$ detection and two independent classification criteria (Cohen's d with 95% CI).

Threshold for AFisomax detection	Algorithm-based classification (n_S = 140, n_U = 71)	Examiners' classification (n_S = 138, n_U = 64)
**Reference (4 / 2 °/s; muscle-specific)**	**6.62 [5.92, 7.31]**	**8.02 [7.19, 8.86]**
Stricter (3 / 1.5 °/s; muscle-specific)	9.00 [8.09, 9.91]	10.01 [8.98, 11.03]
More liberal (5 / 2.5 °/s; muscle-specific)	4.78 [4.24, 5.32]	5.95 [5.29, 6.60]
Uniform 2 °/s (across all muscles)	6.68 [5.98, 7.38]	6.97 [6.23, 7.72]
Uniform 3 °/s (across all muscles)	4.30 [3.80, 4.80]	4.80 [4.24, 5.36]

All p < 0.001. n_S = stable, n_U = unstable muscle groups. Algorithm-based classification uses a least-squares linear fit without intercept (gX = c · F), applied to the gyroscope signal gX (°/s) and the force signal F (N). Examiners' classification is based on direct clinical observation of yielding behaviour during the test and is independent of the algorithmic computation. Both classifications are aggregated from three individual trials per muscle group (n = 230 muscle groups total; reduced n in examiners' classification due to borderline ratings excluded from the stable–unstable comparison).

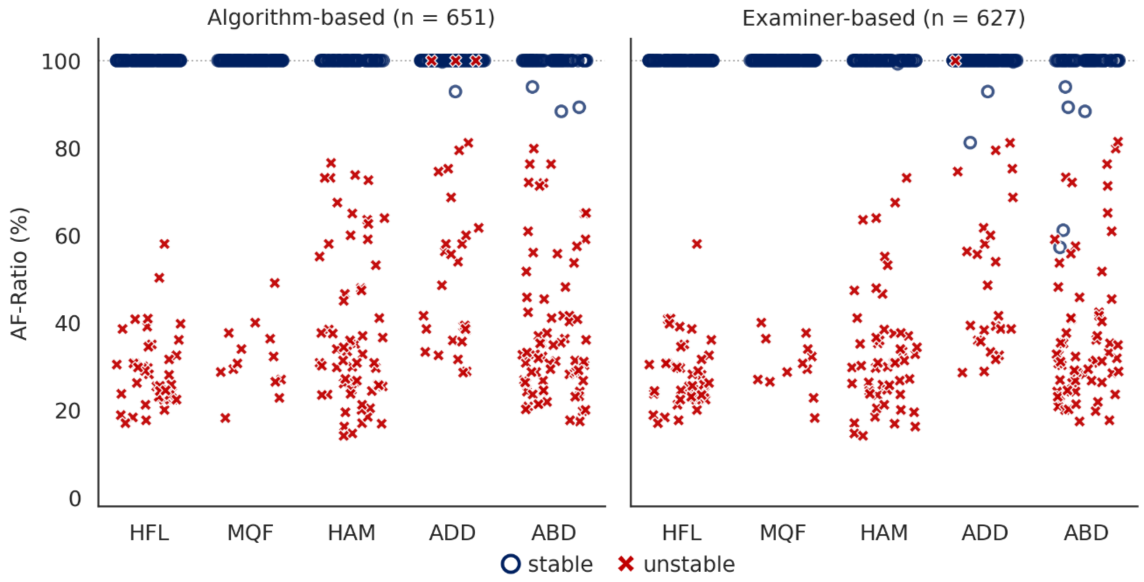

SI Figure 2. Distribution of individual-trial AF-Ratio values by muscle and stability category.

Displayed are the AF-Ratio values of all single trials (n) with definitive stable or unstable classification (borderline not shown). Detection thresholds for AFiso$_{max}$: 4°/s for HFL, MQF and HAM; 2°/s for ADD and ABD.
Left panel: classification by the algorithm; right panel: classification by the examiners.
Blue open circles: trials classified as stable; red crosses: trials classified as unstable.
HFL, hip flexors; MQF, knee extensors; HAM, knee flexors; ADD, hip adductors; ABD, hip abductors.
